# Effect of Hydration on Viscoelastic Tensile Properties of Sclera

**DOI:** 10.3390/vision9010001

**Published:** 2025-01-04

**Authors:** Hamed Hatami-Marbini

**Affiliations:** Mechanical and Industrial Engineering Department, University of Illinois Chicago, Chicago, IL 60607, USA; hatami@uic.edu; Tel.: +1-312-413-2126

**Keywords:** biomechanics, tensile experiments, stress–relaxation, porcine sclera, water content

## Abstract

The present work characterized the effects of hydration on the viscoelastic tensile properties of the sclera. Scleral strips were dissected from the posterior region near the optic nerve head of porcine eyes in the superior–inferior direction. The samples were divided into four hydration groups and their mechanical response was characterized by conducting uniaxial tensile stress–relaxation experiments. An exponential relation and logarithmic expression were used to numerically represent the experimental measurements during the ramp and relaxation periods, respectively. A nonlinear increase in the tensile stress during the ramp period was observed for all strips. Furthermore, it was found that dehydrated specimens had stiffer tensile properties. In particular, it was observed that the maximum and equilibrium stresses increased significantly with decreasing hydration. Furthermore, it was found that the viscoelastic tensile response of porcine scleral strips at different hydration levels could be collapsed onto a single normalized curve. The findings of the present work showed that hydration had significant effects on the viscoelastic tensile properties of sclera.

## 1. Introduction

The sclera is a connective tissue contributing to the structural rigidity of the eyeball. The pathology of eye disorders and diseases such as myopia and glaucoma is attributed to changes in the biomechanical response of sclera. For example, axonal damage occurs in chronic open-angle glaucoma possibly because of injurious stretching of the optic nerve head and lamina cribrosa. The biomechanics of the sclera is believed to play an important role in transferring forces caused by the elevated intraocular pressure to the tissues in the back of the eye [1,2,3]. Furthermore, progressive myopia is accompanied by significant changes in scleral mechanical properties. Specifically, the sclera of myopic eyes has been shown to be soft and creep more [4,5].

The mechanical properties of the sclera depend on the architecture and composition of its extracellular matrix (ECM), which is primarily composed of collagen fibers, elastin, and proteoglycans (PGs) [6]. Collagen fibers, surrounded by PGs, form bundles that are grouped into an extensively interwoven mesh of lamellae within the plane of the sclera. PGs are composed of a core protein to which negatively charged glycosaminoglycan (GAG) side chains are covalently attached [7]. The highly negatively charged glycan chains of PGs induce a strong tendency for the sclera to swell when immersed in aqueous environment. Under the physiological condition, the hydrophilic nature of GAGs and their interaction with other components of the ECM regulate tissue hydration. The hydration and its relation to scleral permeability have been studied in trans-scleral drug delivery investigations [8,9]. Hydration variation has also been shown to affect the molecular structure and biomechanical properties of the corneal and scleral ECM [10,11,12,13]. The present study was conducted to characterize the hydration effects on viscoelastic tensile properties of sclera following the methods that we previously used to determine the effects of hydration on the mechanical properties of cornea [14].

There are clear structural differences between the scleral and corneal ECMs [15]. The sclera has significantly higher collagen content, larger collagen fibers (about 25 nm to 250 nm), lower GAG concentration, and significantly more interweaving and branching of collagen fibers. On the other hand, corneal collagen fibers, with an almost uniform diameter of about 25 nm, are organized in thin lamellae lying on top of each other [16]. Because of its distinctly different microstructure and composition, the sclera swells significantly less than the cornea [10]. Thus, hydration effects on stress–relaxation tensile properties of the sclera are expected to be different and need to be investigated. The present work used the uniaxial tensile testing method to characterize the effect of hydration on the viscoelastic mechanical response of the sclera.

## 2. Materials and Methods

**Figure 1:**
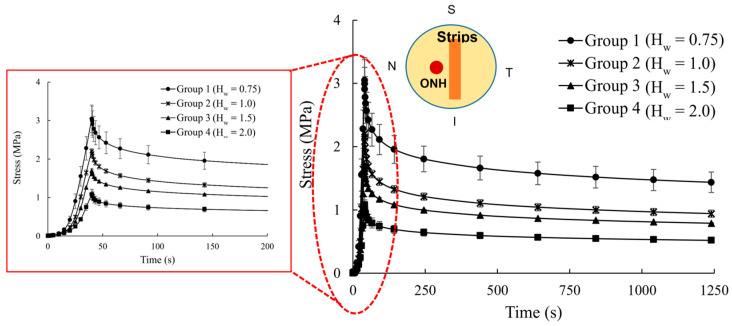
Stress–relaxation behavior of porcine scleral strips with hydration 0.75 (Group 1), 1.0 (Group 2), 1.5 (Group 3), and 2.0 (Group 4) mg water/mg dry tissue. The inset shows the location and direction of extracted strips from porcine eyes. A close-up of the viscoelastic tensile response of specimens from different hydration groups at the early stage of mechanical tests is also shown.

The eyeballs from six- to eight-month-old pigs were obtained from a local slaughterhouse, brought to the laboratory on ice, and were used for mechanical tests the same day. Although the health of animals and their exact age were not known, all eyes were inspected for the presence of scars or any areas of damage. In the case of any physical damage, the eyes were excluded from this study. Before making an incision and extraction of scleral strips, pig eyes were carefully cleaned by removing extraocular fat and muscles from them. Scleral strips with 5 mm × 20 mm dimensions were dissected from the posterior region of *n* = 20 eyeballs. To prevent any regional property variation effects on the experimental measurements [17,18], the strips were only obtained from the superior–inferior direction (Figure 1). After measuring the weight of strips, they were placed in a desiccator filled with silica gels at room temperature for 48 h to dry. A precision analytical scale with 0.1 mg accuracy was used to measure their dry (solid) weight, *W_dry_*. Hydration, *H_w_*, is defined as the amount of water divided by the dry weight of strips. We divided scleral strips equally into four distinct hydration groups, i.e., *H_w_* = 0.75 (Group 1, *n* = 5), 1.0 (Group 2, *n* = 5), 1.5 (Group 3, *n* = 5), and 2.0 (Group 4, *n* = 5) mg water/mg dry tissue by immersing them in PBS until their wet weight, *W_wet_*, reached

(1) $$W_{wet}=\left(1+H_{w}\right)W_{dry}$$

A dynamic mechanical analyzer machine (RSA-G2, TA Instruments) was used to measure viscoelastic tensile properties of scleral strips. The thickness and width of specimens in each hydration group were measured prior to mounting them to the testing machine; these data were used to calculate the initial cross-sectional area. Sandpaper was used at clamps to eliminate slippage. Furthermore, mineral oil was used as the bathing solution to avoid any hydration change during the experiments. The experiments were performed at room temperature, e.g., 22 °C. After straightening the samples by applying a small tensile preload, we used ten ramp loading and unloading cycles to precondition them. At the end of the preconditioning step, the unloaded strips were allowed to rest for five minutes. Then, they were subjected to a preload of *σ_tare_* = 0.01 MPa in order to straighten them and determine their initial length, *L*_0_. The engineering strain *ε* was defined as the ratio of total displacement and the initial length,

*ε* = (*L* − *L*_0_)/*L*_0_ (2)

where *L* is the length of strips. The tensile stress *σ* was obtained from dividing the measured experimental force by the initial cross-sectional area. The viscoelastic stress–relaxation response of the strips was characterized by stretching the samples at a displacement rate of 10 µm/s and then allowing them to relax for 20 min. In addition to the four hydration groups, the stress–relaxation response of eight freshly excised strips, without being initially dried, was determined using oil (n = 4) and PBS (n = 4) as the bathing solution. These mechanical tests were conducted to assess the effects of using mineral oil as the bathing solution on viscoelastic tensile property measurements of sclera [12,19].

The numerical analysis of experimental data was conducted by considering the mechanical behavior of strips during the ramping time and relaxation period separately [20]. In particular, stress–strain and stress–relaxation behaviors were captured, respectively, by

(3) $$\sigma =\alpha \left(e^{\beta \varepsilon }-1\right)+\sigma_{tare}$$

(4) $$\sigma =\gamma -\omega \ln(t-t_{0})$$

where *σ* is the stress, *ε* is the strain, and *t* is time. The fit parameters *α*, *β*, *γ*, and *ω* were computed from curve-fitting the above relations to the experimental measurements using the least-squares optimization technique. The coefficient of determination *R^2^* gave the goodness of the fits. From the experimental measurements, we obtained the maximum stress during the ramping period, the equilibrium stress at the end of the relaxation time, and the maximum tangent modulus, defined as the slope of the stress–strain curve at the maximum strain, which occurred at the end of the ramping time. The above values were reported as the mean ± one standard deviation and were used to statistically compare the viscoelastic tensile response of different hydration groups. The one-way ANOVA with a significance level of 0.05 was used for comparing the viscoelastic tensile response of four hydration groups.

## 3. Results

Figure 1 shows that scleral strips had a significantly stiffer mechanical response as their hydration decreased. A nonlinear increase in the tensile stress was observed during the ramp period in all hydration groups. During the relaxation period, the tensile stress decreased initially at a high rate but reached an almost steady state after 20 min.

**Figure 2:**
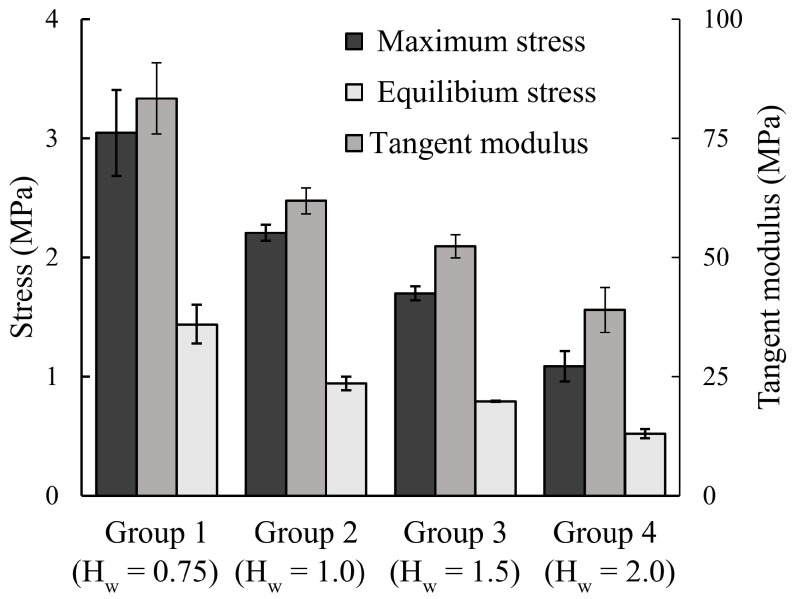
The effect of hydration on the maximum tensile stress, equilibrium stress, and maximum tangent modulus of porcine scleral strips. A significant hydration effect (*p* < 0.05) was observed on all mechanical parameters representing the viscoelastic behavior of different hydration groups.

Figure 2 depicts that there was a significant hydration effect on the maximum stress, equilibrium stress, and maximum tangent modulus (*p* < 0.05), i.e., scleral strips showed significantly stiffer mechanical response with decreasing hydration.

**Figure 3:**
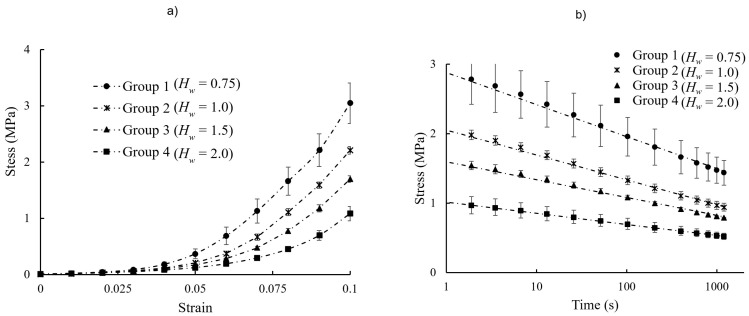
(**a**) Stress–strain and (**b**) stress–relaxation response of porcine scleral strips with different hydration. The experimental measurements were represented numerically by an exponential function and logarithmic mathematical relation. The fit parameters are given in Table 1.

**Figure 4:**
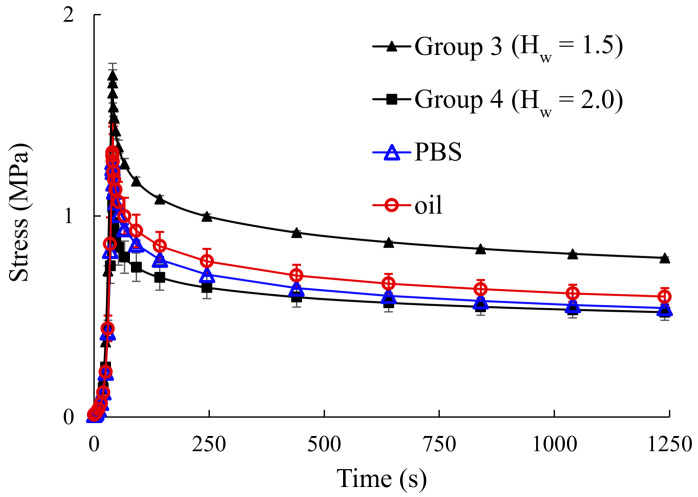
Stress–relaxation behavior of freshly excised scleral strips that were not initially dried. The strips were expected to have hydration of about 1.76 ± 0.10 mg water/mg dry tissue. The experiments were conducted in mineral oil as well as PBS in order to assess the effects of the bathing solution on mechanical measurements.

The stress–strain behavior and stress–relaxation response of different hydration groups, along with their corresponding numerical fits, are shown in Figure 3. Table 1 gives the fit parameters and R^2^ values. Figure 4 shows the stress–relaxation response of freshly excised strips, tested in mineral oil and PBS, and compares the findings with what was obtained for scleral strips in hydration Groups 3 and 4.

## 4. Discussion

Different experimental methods have been used to characterize the mechanical behavior of the sclera without paying attention to the possible effects of hydration [1]. Like many other soft tissues, the architecture and composition of structural proteins constituting the scleral ECM define its biomechanical properties. The ECM of the sclera is primarily composed of collagen, elastin, and proteoglycans (PGs) [6,15]. PGs are macromolecules whose negatively charged glycosaminoglycan (GAG) side chains are dispersed between collagen fibrils. In addition to their important function in collagen fibril organization and fibrogenesis [21,22], PGs/GAGs are responsible for trapping and keeping high amounts of water inside the tissue. The presence of fixed charges, whose interaction with free mobile ions leads to strong osmotic swelling pressure (Gibbs–Donnan effect), creates an innate tendency for the sclera to swell when incubated in an aqueous solution. This fluid absorption could change the organization of ECM ingredients and is thus expected to have important implications for the biomechanical properties of the sclera.

The present study found that scleral strips became stiffer with decreasing hydration, Figure 1 and Figure 2. From the mechanics viewpoint, the ECM of sclera behaves as a composite domain composed of collagen fibril reinforcements embedded in a hydrated PG matrix. As the hydration of the tissue decreases, the free water in the PG matrix is lost and the stiffness of the matrix domain increases [23]. Thus, a significant increase in the overall stiffness of the ECM is expected according to the mixture theory [12,19].

Figure 2 clearly shows that decreasing hydration significantly stiffened the sclera. This finding is important as previous reports suggested that diseased states as well as aging would cause significant changes in tissue hydration and the rate of PG synthesis [24,25,26,27,28]. For example, it has been observed that the concentration of small PGs, e.g., decorin, inside the scleral ECM as well as the hydration of scleral tissue significantly reduce with age [24,26]. Aged scleral samples have also been shown to become stiffer [29]. Thus, it could be hypothesized that aging decreases hydration, which in turn increases scleral rigidity. It is interesting to mention that this hypothesis was first proposed many years ago as a possible explanation for the then-observed significant different tensile response of posterior scleral strips from newborn and adult eyes [30]. The present study confirmed that a significant decrease in hydration would indeed lead to substantial increasing of scleral stiffness. Despite the above discussion, it should be mentioned that it was not the purpose of this study to provide any information on the effects of aging on biomechanical properties of sclera; there exist other important age-related ultrastructural changes to scleral ECM. For instance, the significant accumulation of nonenzymatic glycation-type cross-links during aging should also contribute to the stiffening of the sclera [31]. Furthermore, the hydration difference between old and young sclera is probably related to the changes that might have occurred to the composition and arrangement of ECM constituents during aging and growth [32].

We have recently shown that hydration has a significant effect on the tensile properties of the cornea and sclera [11,19]. The swelling behaviors of the sclera and cornea are very different. The cornea swells significantly in vitro when immersed in PBS [10,33]. The significantly higher hydration of the cornea is possibly because its GAG content is approximately eight times more than that of the sclera [10,34,35]. In addition to a higher concentration of GAGs, corneal collagen fibrils are arranged in thin lamellae lying on top of each other with little interweaving (especially in posterior regions). Thus, very little force opposing the swelling exists, meaning that the presence of an endothelium-based pumping mechanism is an absolute necessity for maintaining corneal hydration in in vivo conditions. Nevertheless, scleral interwoven collagen fibrils, to which the core protein of PGs are covalently attached, limit scleral swelling and act as an opposing force to its swelling tendency. Our previous study characterizing the hydration-dependent response of sclera [11] used stress-controlled tensile measurements and determined the effect of hydration on maximum strain and hysteresis. In the present study, we used stress–relaxation tensile experiments to complement this previous study and determined the effect of hydration on viscoelastic tensile behavior of sclera.

**Figure 5:**
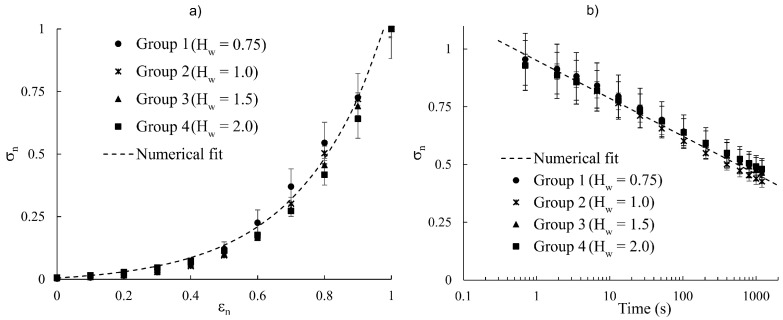
Normalized (**a**) stress–strain behavior and (**b**) normalized stress–relaxation response of different hydration groups. The experimental measurements were successfully collapsed onto a single normalized curve.

The experimental measurements of the present study were represented numerically with an exponential and a logarithmic mathematical function, Equations (3) and (4); *R*^2^ > 99%, Table 1. Considering the scleral behavior during the ramping time and relaxation period separately is an oversimplification and other models, e.g., quasilinear viscoelasticity, are required to capture the constitutive behavior of the sclera [14,36,37]. Such models were not used because it was not the purpose of this study to develop a constitutive model for the viscoelastic time-dependent behavior of the sclera. Figure 5 shows that the stress–strain and stress–relaxation measurements for hydration groups can be collapsed onto normalized curves with respective equations

(5) $$\sigma_{n}\sim 0.02\left(e^{4\varepsilon_{n}}-1\right)$$

(6) $$\sigma_{n}\sim 1-0.7\ln(\varepsilon ),$$

where $\sigma_{n}=\sigma /\sigma (\varepsilon =0.1)$ and $\varepsilon_{n}=\varepsilon /0.1$. The existence of these normalized curves implies that the tensile response of the sclera at any hydration can be mapped to its behavior at a desired hydration using the mathematical relations (5) and (6).

The dehydration of the sclera possibly occurs in two stages [23]. During the first stage, the loss of interfibrillar water occurs without any effect on the diameter of collagen fibers. In the second stage, collagen fibrils become dehydrated, a process that is accompanied by significant structural changes to collagen fibrils and their diameter. Based on the above theory, we believe that the present work only investigated the first stage of scleral dehydration. This is because the experimental measurements from different hydration groups could be collapsed onto a single normalized curve. In this study, a maximum hydration of 2.0 mg water/dry tissue was considered primarily because the rate of swelling decreased significantly as the hydration of samples reached about 2.0 mg water/mg dry tissue [11]. Furthermore, the hydration of posterior scleral samples immediately after dissection was about 1.76 ± 0.10 mg water/mg dry tissue, which is comparable with what was obtained in a previous study [38]. The slight variation between different studies could be because different samples and techniques were used. Furthermore, the hydration might be non-uniform in the posterior region of porcine sclera because of regional thickness variations [18,39].

In a previous study, the thickness of corneal samples, along with a thickness–hydration relation, was used to divide samples into different hydration groups [12]. Thus, samples within a group may not have had exactly similar hydration. The present work avoided this drawback by using hydration instead of thickness to divide specimens into different groups. However, drying and rehydrating samples may have affected the experimental measurements. In order to investigate the possible effects of the drying and rehydrating step, samples were also tested using the same protocol immediately after they had been excised from eyeballs (Figure 4). It was seen that the mechanical response of these specimens agreed well with the findings for those that had undergone the dehydration and hydration cycle. For example, as shown in Figure 4, the mechanical response of freshly excised strips was between what was obtained for hydration Groups 3 and 4. This observation is because these specimens had an average hydration of less than 2.0 mg water/mg dry tissue and greater than 1.5 mg water/mg dry tissue. The hydration of freshly dissected strips was not known since they were not initially dried. However, their hydration is expected to be about 1.76 ± 0.10 mg water/mg dry tissue, which was the average post-dissection hydration of scleral strips in the four hydration groups of the present study. Figure 4 also shows that using mineral oil as the bathing solution did not significantly influence the mechanical measurements. The slight difference between the measured properties when samples tested in mineral oil and PBS could be because the samples continued to swell in PBS, causing their equilibrium mechanical response to become more similar to that of samples in the hydration Group 4. Finally, it noted that the tangent modulus (35 MPa–55 MPa) found here for hydration Groups 3 and 4 and freshly dissected strips was in an overall agreement with previous reports [40,41]. For example, Geraghty et al. reported a maximum tangent modulus of about 40 MPa–52 MPa for porcine sclera, prepared from the superior sector, when tested in Eusol-C solution one day post-mortem. This overall agreement between the results is because samples in these previous studies were tested when their hydration was within the range of the hydration of Groups 3 and 4.

The present study did not quantify the influence of hydration on the human scleral samples. However, because of similarities between human and porcine scleral tissue [42], it is expected that hydration would have a similar effect on the viscoelastic tensile behavior of human posterior sclera. Despite this, the amount of hydration effects is expected to be different because human sclera was found to be stiffer than porcine sclera [41]. The uniaxial tensile testing method has a number of well-known drawbacks and does not necessarily measure biomechanical properties under physiological conditions [19]. This testing technique does not subject scleral samples to in vivo biaxial loading. Furthermore, initially curved strips need to be straightened prior to the mechanical tests. A certain level of microstructural remodeling is also possible because of the preconditioning step. Thus, this testing technique does not necessarily represent true scleral mechanical properties under physiological conditions. Despite these and other limitations of the strip extensometry, this experimental method is an excellent tool for conducting comparative studies in which possible effects of a parameter on the mechanical response are sought. Using this method, the present work confirmed that hydration played an important role in defining tensile properties of sclera. Thus, hydration of scleral samples is required to be controlled in mechanical property characterization experiments.

## Figures and Tables

**Table 1:** The thickness, maximum tangent modulus, equilibrium stress, maximum stress, and fit parameters for different groups.

					σ = α (e^βε^ − 1) + σ_0_			σ = γ − ω ln(t − t_0_)	
Group	Thickness (μm)	E (MPa)	σ_eq_ (MPa)	σ_max_ (MPa)	α (MPa)	β	σ_0_ (KPa)	γ (MPa)	ω (MPa)
1	700 ± 160	83.3 ± 7.5	3.1 ± 0.8	1.4 ± 0.4	0.11 ± 0.09	35.9 ± 6.0	8.4 ± 0.7	2.9 ± 0.9	0.20 ± 0.07
2	750 ± 180	61.9 ± 2.8	2.2 ± 0.2	0.9 ± 0.1	0.05 ± 0.02	39.90 ± 4.1	9.5 ± 0.2	2.1 ± 0.2	0.16 ± 0.01
3	1110 ± 190	52.3 ± 2.4	1.7 ± 0.1	0.8 ± 0.0	0.03 ± 0.01	41.90 ± 2.4	9.3 ± 0.7	1.6 ± 0.1	0.11 ± 0.02
4	1270 ± 140	40.0 ± 4.8	1.1 ± 0.3	0.5 ± 0.1	0.02 ± 0.00	42.00 ± 4.2	7.8 ± 0.4	1.0 ± 0.3	0.07 ± 0.03

## Data Availability

The original contributions presented in this study are included in the article. Further inquiries can be directed to the corresponding author.

## References

[1] Campbell I.C., Coudrillier B., Ethier C.R. (2014). Biomechanics of the posterior eye: A critical role in health and disease. J. Biomech. Eng..

[2] Coudrillier B., Pijanka J.K., Jefferys J.L., Goel A., Quigley H.A., Boote C., Nguyen T.D. (2015). Glaucoma-related changes in the mechanical properties and collagen micro-architecture of the human sclera. PLoS ONE.

[3] Pijanka J.K., Coudrillier B., Ziegler K., Sorensen T., Meek K.M., Nguyen T.D., Quigley H.A., Boote C. (2012). Quantitative mapping of collagen fiber orientation in non-glaucoma and glaucoma posterior human sclerae. Investig. Ophthalmol. Vis. Sci..

[4] Phillips J., McBrien N. (1995). Form deprivation myopia: Elastic properties of sclera. Ophthalmic Physiol. Opt..

[5] McBrien N.A., Jobling A.I., Gentle A. (2009). Biomechanics of the sclera in myopia: Extracellular and cellular factors. Optom. Vis. Sci..

[6] Watson P.G., Young R.D. (2004). Scleral structure, organisation and disease. A review. Exp. Eye Res..

[7] Heinegård D. (2009). Fell-Muir Lecture: Proteoglycans and more—From molecules to biology. Int. J. Exp. Pathol..

[8] Boubriak O., Urban J., Akhtar S., Meek K., Bron A. (2000). The effect of hydration and matrix composition on solute diffusion in rabbit sclera. Exp. Eye Res..

[9] Ambati J., Gragoudas E.S., Miller J.W., You T.T., Miyamoto K., Delori F.C., Adamis A.P. (2000). Transscleral Delivery of Bioactive Protein to the Choroid and Retina. Investig. Ophthalmol. Vis. Sci..

[10] Huang Y., Meek K.M. (1999). Swelling studies on the cornea and sclera: The effects of pH and ionic strength. Biophys. J..

[11] Hatami-Marbini H., Pachenari M. (2020). Hydration related changes in tensile response of posterior porcine sclera. J. Mech. Behav. Biomed. Mater..

[12] Hatami-Marbini H., Rahimi A. (2014). The relation between hydration and mechanical behavior of bovine cornea in tension. J. Mech. Behav. Biomed. Mater..

[13] Hatami-Marbini H., Etebu E. (2013). Hydration dependent biomechanical properties of the corneal stroma. Exp. Eye Res..

[14] Hatami-Marbini H. (2014). Hydration dependent viscoelastic tensile behavior of cornea. Ann. Biomed. Eng..

[15] Meek K.M., Fratzl P. (2008). The Cornea and Sclera. Collagen: Structure and Mechanics.

[16] Komai Y., Ushiki T. (1991). The three-dimensional organization of collagen fibrils in the human cornea and sclera. Investig. Ophthalmol. Vis. Sci..

[17] Elsheikh A., Geraghty B., Alhasso D., Knappett J., Campanelli M., Rama P. (2010). Regional variation in the biomechanical properties of the human sclera. Exp. Eye Res..

[18] Hatami-Marbini H., Mehr J.A. (2024). Regional differences in electroactive response of the sclera. Proc. Inst. Mech. Engineers. Part H J. Eng. Med..

[19] Hatami-Marbini H., Rahimi A. (2014). Effects of bathing solution on tensile properties of the cornea. Exp. Eye Res..

[20] Hatami-Marbini H., Rahimi A. (2015). Evaluation of hydration effects on tensile properties of bovine corneas. J. Cataract. Refract. Surg..

[21] Meek K.M., Fullwood N.J. (2001). Corneal and scleral collagens—A microscopist’s perspective. Micron.

[22] Birk D.E., Fitch J.M., Babiarz J.P., Doane K.J., Linsenmayer T.F. (1990). Collagen fibrillogenesis in vitro: Interaction of types I and V collagen regulates fibril diameter. J. Cell Sci..

[23] Fratzl P., Daxer A. (1993). Structural transformation of collagen fibrils in corneal stroma during drying. An x-ray scattering study. Biophys. J..

[24] Brown C., Vural M., Johnson M., Trinkaus-Randall V. (1994). Age-related changes of scleral hydration and sulfated glycosaminoglycans. Mech. Ageing Dev..

[25] Harper A.R., Summers J.A. (2015). The dynamic sclera: Extracellular matrix remodeling in normal ocular growth and myopia development. Exp. Eye Res..

[26] A Rada J., Achen V.R., Penugonda S., Schmidt R.W., A Mount B. (2000). Proteoglycan composition in the human sclera during growth and aging. Investig. Ophthalmol. Vis. Sci..

[27] Rada J.A.S., Shelton S., Norton T.T. (2006). The sclera and myopia. Exp. Eye Res..

[28] Rada J.A., Nickla D.L., Troilo D. (2000). Decreased proteoglycan synthesis associated with form deprivation myopia in mature primate eyes. Investig. Ophthalmol. Vis. Sci..

[29] Coudrillier B., Pijanka J., Jefferys J., Sorensen T., Quigley H.A., Boote C., Nguyen T.D. (2015). Effects of age and diabetes on scleral stiffness. J. Biomech. Eng..

[30] Curtin B.J. (1969). Physiopathologic aspects of scleral stress-strain. Trans. Am. Ophthalmol. Soc..

[31] Paul R.G., Bailey A.J. (1996). Glycation of collagen: The basis of its central role in the late complications of ageing and diabetes. Int. J. Biochem. Cell Biol..

[32] Coudrillier B., Pijanka J., Jefferys J., Sorensen T., Quigley H.A., Boote C., Nguyen T.D. (2015). Collagen structure and mechanical properties of the human sclera: Analysis for the effects of age. J. Biomech. Eng..

[33] Hatami-Marbini H., Etebu E., Rahimi A. (2013). Swelling pressure and hydration behavior of porcine corneal stroma. Curr. Eye Res..

[34] Hatami-Marbini H. (2023). On the mechanical roles of glycosaminoglycans in the tensile properties of porcine corneal stroma. Investig. Ophthalmol. Vis. Sci..

[35] Hatami-Marbini H., Pachenari M. (2020). On influence of sulfated glycosaminoglycans on tensile properties of posterior sclera. Mech. Soft Mater..

[36] Fung Y. (2013). Biomechanics: Mechanical Properties of Living Tissues.

[37] Hatami-Marbini H., Maulik R. (2016). A Biphasic Transversely Isotropic Poroviscoelastic Model for the Unconfined Compression of Hydrated Soft Tissue. J. Biomech. Eng..

[38] Murienne B.J., Jefferys J.L., Quigley H.A., Nguyen T.D. (2015). The effects of glycosaminoglycan degradation on the mechanical behavior of the posterior porcine sclera. Acta Biomater..

[39] Norman R.E., Flanagan J.G., Rausch S.M., Sigal I.A., Tertinegg I., Eilaghi A., Portnoy S., Sled J.G., Ethier C.R. (2010). Dimensions of the human sclera: Thickness measurement and regional changes with axial length. Exp. Eye Res..

[40] Geraghty B., Jones S.W., Rama P., Akhtar R., Elsheikh A. (2012). Age-related variations in the biomechanical properties of human sclera. J. Mech. Behav. Biomed. Mater..

[41] Schultz D.S., Lotz J.C., Lee S.M., Trinidad M.L., Stewart J.M. (2008). Structural factors that mediate scleral stiffness. Investig. Ophthalmol. Vis. Sci..

[42] Nicoli S., Ferrari G., Quarta M., Macaluso C., Govoni P., Dallatana D., Santi P. (2009). Porcine sclera as a model of human sclera for in vitro transport experiments: Histology, SEM, and comparative permeability. Mol. Vis..

